# Cross-Cultural Adaptation and Psychometric Validation of the Greek Version of the Swallowing Disturbance Questionnaire (g-SDQ) in Persons with Multiple Sclerosis

**DOI:** 10.3390/healthcare14213710

**Published:** 2026-11-02

**Authors:** Nefeli K. Dimitriou, Christos Bakirtzis, Georgios Dimakopoulos, Angeliki Alivizatou, Grigorios Nasios

**Affiliations:** 1 Department of Speech and Language Therapy, University of Ioannina, 45500 Ioannina, Greece; nasios@uoi.gr; 2 Second Department of Neurology, School of Medicine, Aristotle University of Thessaloniki, 54124 Thessaloniki, Greece; cbakirtzis@auth.gr; 3 BIOSTATS, Science and Technology Park of Epirus, 45500 Ioannina, Greece; info@biostats.gr; 4 Department of Medicine, University of Thessaly, 41500 Larisa, Greece; aggeliki.alivizatou@gmail.com

**Keywords:** multiple sclerosis, dysphagia, swallowing disturbance questionnaire, screening, validation, patient-reported outcome measure

## Abstract

Background/Objectives: Dysphagia affects approximately 45% of persons with multiple sclerosis (pwMS) and is a major cause of morbidity and mortality. The present study aimed to translate, cross-culturally adapt, and psychometrically validate the Greek version of the Swallowing Disturbance Questionnaire (g-SDQ) in pwMS. Methods: Eighty-eight adults with clinically stable MS completed the g-SDQ together with the Dysphagia in Multiple Sclerosis (DYMUS) questionnaire, the Speech Pathology-Specific Questionnaire for Persons with Multiple Sclerosis (SMS), the Brief International Cognitive Assessment for Multiple Sclerosis (BICAMS) battery, and Verbal Fluency (VF) tests. All participants also underwent the 3-ounce Water Swallow Test (3oz-WST). Results: The g-SDQ showed good internal consistency (α = 0.857). Principal component analysis, supported by parallel analysis, yielded a two-component solution explaining 51.1% of the variance, although the components did not reproduce the theoretical oral/pharyngeal division. Screening discrimination against the 3oz-WST was high (AUC = 0.912, 95% CI 0.791–0.990); a cut-off of ≥6.5 yielded a sensitivity of 85.7% and a specificity of 93.2%, whereas the previously proposed cut-off of ≥11 detected only 50.0% of WST-positive participants. After false discovery rate (FDR) correction, the g-SDQ correlated significantly with the DYMUS (ρ =0.722), the SMS (ρ = 0.722), the EDSS (ρ = 0.250), and phonemic VF (ρ = −0.301), whereas associations with the SDMT, the GVLT-II, and categorical VF were weak and non-significant. Conclusions: The g-SDQ is a brief, non-invasive, and promising tool for screening dysphagia in Greek-speaking pwMS, although its cut-off and dimensional structure require confirmation in larger, independent samples.

## 1. Introduction

Multiple Sclerosis (MS) is a chronic, immune-mediated, and neurodegenerative disease of the central nervous system characterized by inflammation, demyelination, and axonal loss [1,2,3]. The disease course is unpredictable and it is typically classified into three types: relapsing-remitting MS (RRMS), secondary progressive MS (SPMS), and primary progressive MS (PPMS). RRMS is the most common type, with neurologic symptoms appearing suddenly (relapse), followed by a period of complete or near-complete recovery (remission). Over time, relapsing-remitting episodes may occur less frequently and individuals may develop gradually accumulating disability (SPMS). Individuals with PPMS present with progressive accumulation of disability from the onset of the disease [4]. The clinical presentation is highly heterogeneous, encompassing motor deficits, sensory disturbances, visual impairments, autonomic dysfunction, fatigue, depression, cognitive decline, speech and language deficits, communication changes, and swallowing difficulties [5,6,7,8,9].

In particular, dysphagia occurs in 45% of persons with MS (pwMS), a prevalence markedly higher than that of the general population [10]. Swallowing difficulties can arise at any disease stage; however, their frequency rises with age, disease duration, and progressive phenotypes. The brainstem, cerebellum, and corticobulbar pathways are all thought to be involved in its pathogenesis [11]. Disturbances typically involve the oral and pharyngeal phases of swallowing, although upper-oesophageal sphincter dysfunction has also been reported [12]. Dysphagia can lead to several complications, including aspiration pneumonia, malnutrition, and dehydration [13], which reduce quality of life and are common causes of morbidity and death in late stages of MS [14,15]. Nevertheless, its management remains a daily-care challenge, and early diagnosis is crucial [16].

The assessment of dysphagia typically combines medical history, clinical evaluation, and instrumental examinations such as fibreoptic endoscopic evaluation of swallowing (FEES), videofluoroscopic swallowing study (VFSS), and electromyography [17]. Although accurate diagnosis during the early stages is critical to prevent life-threatening aspiration pneumonia [18], these instrumental techniques are rarely used in the initial phases since they are resource-intensive, invasive, and highly stressful for frail patients. Consequently, there is growing reliance on Patient-Reported Outcome Measures (PROMs) as accessible, early screening tools for swallowing difficulties [19].

To this end, tools like the Dysphagia in Multiple Sclerosis (DYMUS) questionnaire have been developed to capture patient-perceived swallowing difficulties in pwMS. However, these PROMs have notable limitations; they only allow for dichotomous responses (yes/no) without staging symptom frequency, and they fail to address dysphagia for saliva or for different food textures such as pureed food [20]. There is, therefore, a clear clinical need for complementary PROMs. The Swallowing Disturbance Questionnaire (SDQ) is a 15-item PROM designed to assess both oral and pharyngeal symptoms, as well as recent respiratory events related to swallowing [21].

Although the SDQ has demonstrated excellent psychometric properties and diagnostic accuracy in pwMS globally [20,21], a validated version optimized for Greek-speaking individuals has yet to be developed. Given that MS affects millions of individuals worldwide [22] and exhibits a substantial and growing prevalence in Greece (estimated at 197.8 per 100,000; 95% CI 197.6–198.0) [23], this omission is critical. The present study therefore aimed to translate, culturally adapt, and psychometrically validate the SDQ in Greek (g-SDQ) in a sample of pwMS.

## 2. Materials and Methods

### 2.1. Participants

The study sample consisted of individuals diagnosed with MS. Demographic (sex, age, and years of education) and clinical (disease duration, MS phenotype, and disability status) characteristics were recorded. Participants were included if they met the following criteria: (1) diagnosis of MS according to the revised 2024 McDonald criteria; (2) age ≥ 18 years; and (3) stable disease, defined as the absence of clinical and radiological activity or disease progression during the 6 months prior to participation. Individuals were excluded if they presented with: (1) concomitant acute psychiatric conditions or severe substance abuse; (2) other neurological disorders (e.g., epilepsy, stroke, Parkinson’s disease, traumatic brain injury); (3) structural or malignant abnormalities of the larynx, pharynx, or upper gastrointestinal tract; or (4) severe visual or auditory impairments.

No *a priori* sample size calculation was performed; all eligible patients seen during the recruitment period were enrolled, yielding a sample comparable to those of the original (*n* = 57) [24] and Italian MS (*n* = 84) [20] SDQ validation studies.

### 2.2. Measures

#### 2.2.1. The Greek Version of the Swallowing Disturbance Questionnaire (g-SDQ)

The g-SDQ is a 15-item PROM that assesses symptoms of dysphagia at any stage of swallowing: items 1–5 pertain to oral-stage symptoms, while items 6–15 concern the pharyngeal phase. Items 1–14 are scored on a 4-point Likert scale reflecting symptom frequency: 0—‘never’; 1—‘rarely’ (≥once per month); 2– ‘often’ (1–7 times per week); and 3—‘very often’ (>7 times per week). Item 15 records recent respiratory infections with a “yes” or “no” response, scored as 2.5 or 0.5, respectively. Higher scores reflect greater subjective dysphagia severity, and a total score ≥11 has been proposed to indicate probable dysphagia [21].

The original English version was initially translated into Greek independently by two bilingual health professionals (a speech-language pathologist and a neurologist) who synthesized a consensus draft. Next, an independent clinician (speech-language pathologist) blinded to the original scale performed a back-translation into English. An expert multidisciplinary committee reviewed all versions to ensure semantic, idiomatic, experiential, and conceptual equivalence; all 15 items were retained without modification. Finally, the Greek version (g-SDQ) underwent pilot testing with 10 healthy controls to confirm comprehensibility, readability, and face validity—unaffected by MS-related fatigue or cognitive symptoms—prior to clinical administration. No major discrepancies emerged during the forward and back-translation steps, and pilot participants reported no difficulty in understanding any item. This procedure is consistent with internationally recommended guidelines for the cross-cultural adaptation of self-report measures [25].

#### 2.2.2. Assessment of the Psychometric Properties of the Scale

To evaluate construct and criterion validity, all participants completed two PROMs previously validated in Greek. The DYMUS [26] is a 10-item yes/no instrument that distinguishes dysphagia for solids and liquids, with an aggregate score from 0 to 10. The Speech Pathology-Specific Questionnaire for Persons with Multiple Sclerosis (SMS) [27] consists of 16 items examining speech/language, voice, and swallowing abilities in pwMS, scored on a 5-point Likert scale with a maximum total score of 64.

Participants also underwent the clinical, non-invasive 3-ounce Water Swallow Test (3oz-WST) [28] as a clinical screening criterion for assessing criterion-related validity. PwMS were instructed to drink 90 mL of water continuously from a cup in their usual manner while the clinician monitored for signs of aspiration, including coughing, choking, changes in voice quality, and post-swallowing respiratory distress. The test outcome was classified as either normal (0) or impaired (1). Overall, these measurements, along with internal consistency and structural validity analyses, formed the comprehensive psychometric evaluation of the g-SDQ.

#### 2.2.3. Cognitive and Language Assessment

Cognitive assessment was conducted both to investigate construct validity (exploring potential associations between subjective swallowing complaints and cognitive performance) and to ensure that cognitive deficits did not compromise participants’ ability to accurately complete self-report instruments.

Cognitive function was evaluated with the Brief International Cognitive Assessment for Multiple Sclerosis (BICAMS; [29], comprising: (1) the Symbol Digit Modalities Test (SDMT), assessing information processing speed, visual scanning, and working memory; (2) the Greek Verbal Learning Test (GVLT), assessing verbal memory; and (3) the Brief Visuospatial Memory Test-Revised (BVMT-R), assessing visual/spatial memory.

The Verbal Fluency (VF) test was also administered to evaluate the participants’ ability to generate words within a limited timeframe. It comprises two components: phonemic VF (phVF), requiring words beginning with a specific letter, and categorical VF (cVF), requiring words from a designated category. VF taps multiple domains including semantic memory, language, attention, processing speed, and executive functions such as inhibition, self-monitoring, and cognitive flexibility [30].

### 2.3. Procedure

The study was conducted in accordance with the Declaration of Helsinki and was approved by the ethics committee of the University of Ioannina (reference number 48936/17-12-2024). Participants were consecutively recruited between December 2024 and February 2026 from an outpatient neurological clinic and a specialized rehabilitation center in Greece. All eligible individuals meeting the diagnostic and clinical criteria were invited to participate during their routine follow-up visits. All participants were informed of the study’s objectives and procedures and provided written informed consent prior to participation. Tasks were carried out in a quiet room to minimize external distractions, with no other individuals present. Questionnaires were administered in a fixed, pre-determined order.

### 2.4. Statistical Analysis

Descriptive statistics were computed for all variables. Distributional assumptions were examined with the Shapiro–Wilk test and by inspection of skewness and kurtosis. Because the g-SDQ total score was markedly non-normal and right-skewed, most clinical comparators also deviated from normality, and the EDSS is an ordinal scale (Section 3.2 and Section 3.6), non-parametric methods were used for inferential analyses. Floor and ceiling effects were considered present when more than 15% of participants obtained the lowest (0.5) or the highest possible (44.5) total score, respectively [31].

Internal consistency was examined with Cronbach’s α, with 95% confidence intervals (CIs) computed by the Feldt method [32], together with corrected item–total correlations and α-if-item-deleted values; α ≥ 0.70 was considered acceptable and α ≥ 0.80 good [31]. α was computed for the total scale, for the theoretical oral (items 1–5) and pharyngeal (items 6–14) subscales, and for each empirically derived component.

Structural validity was explored with principal component analysis (PCA) of the inter-item correlation matrix. Sampling adequacy was assessed with the Kaiser–Meyer–Olkin (KMO) measure (values ≥ 0.60 considered acceptable) and Bartlett’s test of sphericity [33]. Because the eigenvalue > 1 rule tends to over-extract, the number of components to retain was determined with Horn’s parallel analysis (2000 random column-wise permutations of the observed data; components were retained when the observed eigenvalue exceeded the 95th percentile of the permuted eigenvalues) [34], supplemented by Velicer’s minimum average partial (MAP) test [35]. Retained components were subjected to Varimax rotation, and loadings ≥ 0.40 were considered salient; communalities and the full rotated loading matrix are reported. As a sensitivity analysis, a common-factor solution (principal axis factoring with oblique oblimin rotation) was also estimated.

Criterion-related validity against the 3oz-WST (screening discrimination) was assessed by receiver operating characteristic (ROC) analysis. The 95% CI of the area under the curve (AUC) was obtained from 2000 bootstrap resamples (percentile method); AUC values of 0.70–0.80, 0.80–0.90, and ≥ 0.90 were interpreted as acceptable, excellent, and outstanding discrimination, respectively [36]. The optimal cut-off was identified with Youden’s index [37], and sensitivity, specificity, and positive (PPV) and negative (NPV) predictive values were reported with exact Clopper–Pearson 95% CIs [38], together with positive and negative likelihood ratios (LR+, LR−). The previously proposed threshold of ≥11 [21] was evaluated in the same way. Because the cut-off was derived and evaluated in the same dataset, its optimism was estimated by bootstrap internal validation (2000 resamples): in each resample, the Youden-optimal cut-off was re-derived, its performance in the resample was compared with its performance in the original sample, and the mean difference was subtracted from the apparent estimates [39]. The distribution of the selected cut-off across resamples was used to describe its stability.

Convergent and construct validity were examined with Spearman’s rank correlation coefficients (ρ) and 95% CIs based on the Fisher z-transformation with the Bonett–Wright standard error [40]; coefficients < 0.30, 0.30–0.50, and >0.50 were interpreted as weak, moderate, and strong, respectively [41]. To account for multiplicity across the ten correlations, *p*-values were adjusted with the Benjamini–Hochberg false discovery rate (FDR) procedure [42]; Pearson’s r is reported as a sensitivity analysis. g-SDQ scores of WST-positive and WST-negative participants were compared with the Mann–Whitney U test, with r = Z/√N as the effect size.

Post hoc, with 14 WST-positive and 74 WST-negative participants, the study had >95% power (two-sided α = 0.05) to detect an AUC of 0.80 against the null value of 0.50 [43], and the total sample of 88 provided approximately 80% power to detect correlations of |ρ| ≥ 0.30. The participant-to-item ratio (5.9:1) is, however, below commonly recommended ratios for factor-analytic studies, although the adequacy of a given sample size also depends on communalities and factor overdetermination [44]; the structural analyses were therefore regarded as exploratory.

Two-sided *p*-values < 0.05 were considered statistically significant. Analyses were conducted with IBM SPSS Statistics for Windows, Version 29.0 (IBM Corp., Armonk, NY, USA); parallel analysis, the MAP test, bootstrap procedures, and confidence intervals were computed in R (R Core Team, Vienna, Austria).

## 3. Results

### 3.1. Study Population

The final sample comprised 88 PwMS (69.3% female). Mean age was 49.4 years (SD 11.75) and mean educational level was 13.6 years (SD 3.09). Mean disease duration, available for 77 participants, was 15.4 years (SD 8.88). Median Expanded Disability Status Scale (EDSS) was 3.0 (range 1.0–8.0). Regarding phenotype, 61 participants (69.3%) were diagnosed with RRMS, 8 (9.1%) with SPMS, and 0 with PPMS; however, phenotype data were unavailable for 19 participants (21.6%). Fourteen participants (15.9%) had a positive 3oz-WST, consistent with the lower end of the reported prevalence range for dysphagia in MS clinical samples.

### 3.2. SDQ Frequencies

The mean total SDQ score was 3.27 (SD ± 4.30; median 0.5; range 0.5–20.5). The distribution was markedly right-skewed (skewness = 1.73; Shapiro–Wilk W = 0.70, *p*< 0.001). A substantial floor effect was observed: 46 participants (52.3%) obtained the minimum possible score of 0.5 (60.8% of WST-negative participants), whereas no participant reached the maximum possible score of 44.5 (ceiling effect 0%). At the item level, between 73.9% (SDQ10) and 98.9% (SDQ8) of participants scored 0. The frequency of positive responses (score ≥ 1) for each SDQ item is reported in Table 1.

### 3.3. Internal Consistency

Cronbach’s α for the g-SDQ was 0.857 (95% CI 0.809–0.897), indicating good internal consistency. Corrected item-total correlations and α-if-item-deleted values are shown in Table 2. All items exhibited positive corrected item-total correlations, ranging from 0.279 (SDQ14) to 0.722 (SDQ2). No item removal produced a meaningful increase in α (maximum change: 0.000, for SDQ14).

### 3.4. Criterion-Related Validity Against the 3oz-WST

**Figure 1:**
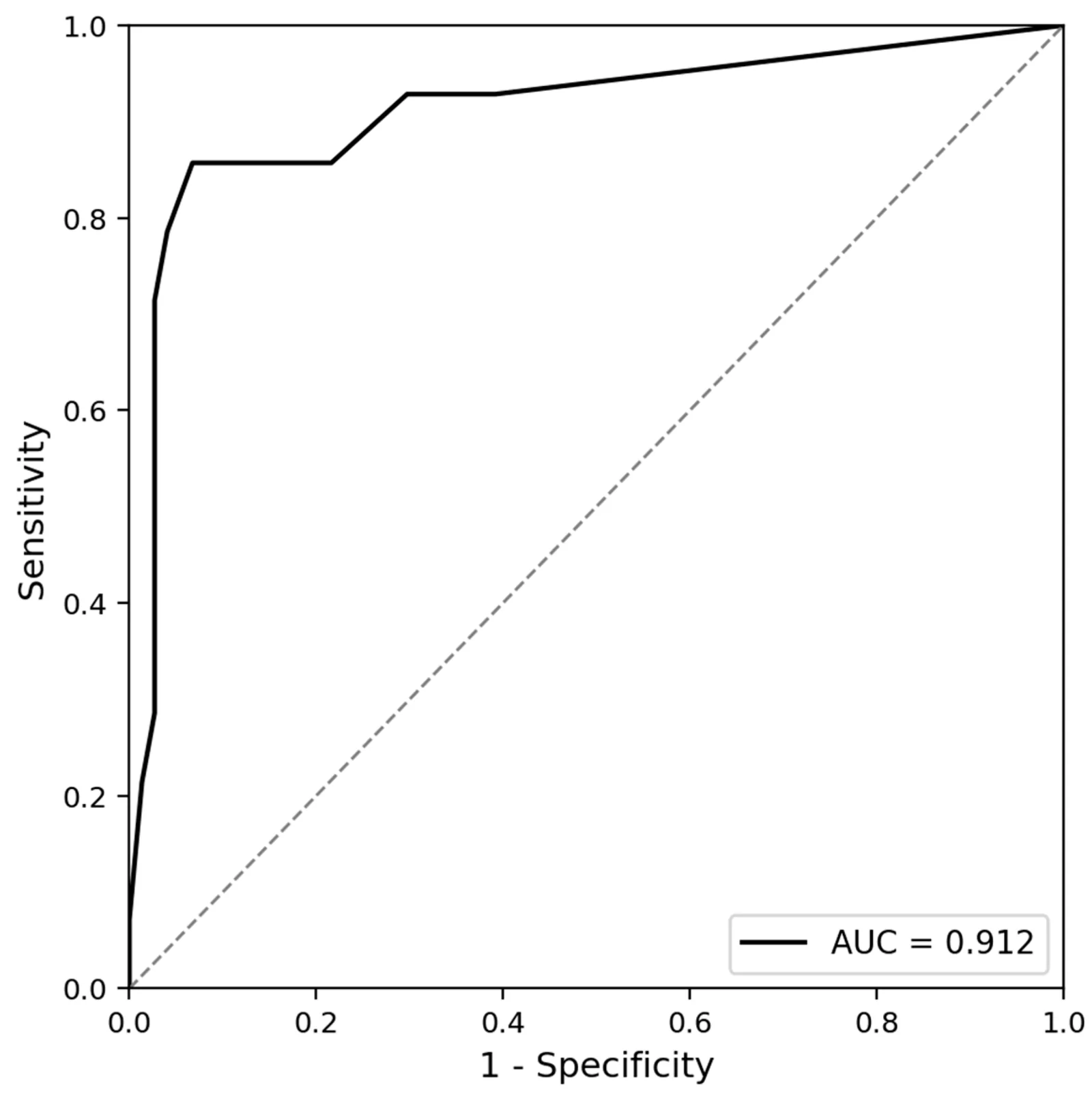
ROC curve for the 15-item g-SDQ.

ROC analysis using the 3oz-WST as the criterion yielded an AUC of 0.912 (95% CI 0.791–0.990; Figure 1), indicating high screening discrimination of the g-SDQ against the 3oz-WST. The optimal cut-off, determined by Youden’s index, was ≥6.5, corresponding to a sensitivity of 85.7% (12/14; 95% CI 57.2–98.2%), a specificity of 93.2% (69/74; 84.9–97.8%), a PPV of 70.6% (44.0–89.7%), and a NPV of 97.2% (90.2–99.7%) (Table 3). Bootstrap internal validation indicated modest optimism (0.041 for sensitivity and 0.001 for specificity), yielding optimism-corrected estimates of 81.6% and 93.2%, respectively. However, the selected cut-off varied across resamples: ≥6.5 was optimal in 59.5% of resamples, ≥8.5 in 23.9%, and ≥9.5 in 11.6% (95% interval 2.5–9.5). Because all total scores end in 0.5, the previously proposed threshold of ≥11 [21] is equivalent to ≥11.5 in practice; in the present cohort, it yielded a sensitivity of 50.0% (7/14; 23.0–77.0%) and a specificity of 97.3% (72/74; 90.6–99.7%) (Table 3).

Applying the risk-stratification approach used by Sparaco et al. [20] in the Italian MS validation (low: score ≤ 3; intermediate: score 4–8; high: score ≥ 9), 60 participants (68.2%) fell in the low-risk category, 14 (15.9%) in the intermediate range, and 14 (15.9%) in the high-risk category. Among participants with a positive 3oz-WST, 78.6% were classified as high-risk, 7.1% as intermediate, and only 14.3% as low-risk. Only one WST-positive participant (7.1%) obtained the minimum g-SDQ score of 0.5.

### 3.5. Structural Validity (Principal Component Analysis)

**Figure 2:**
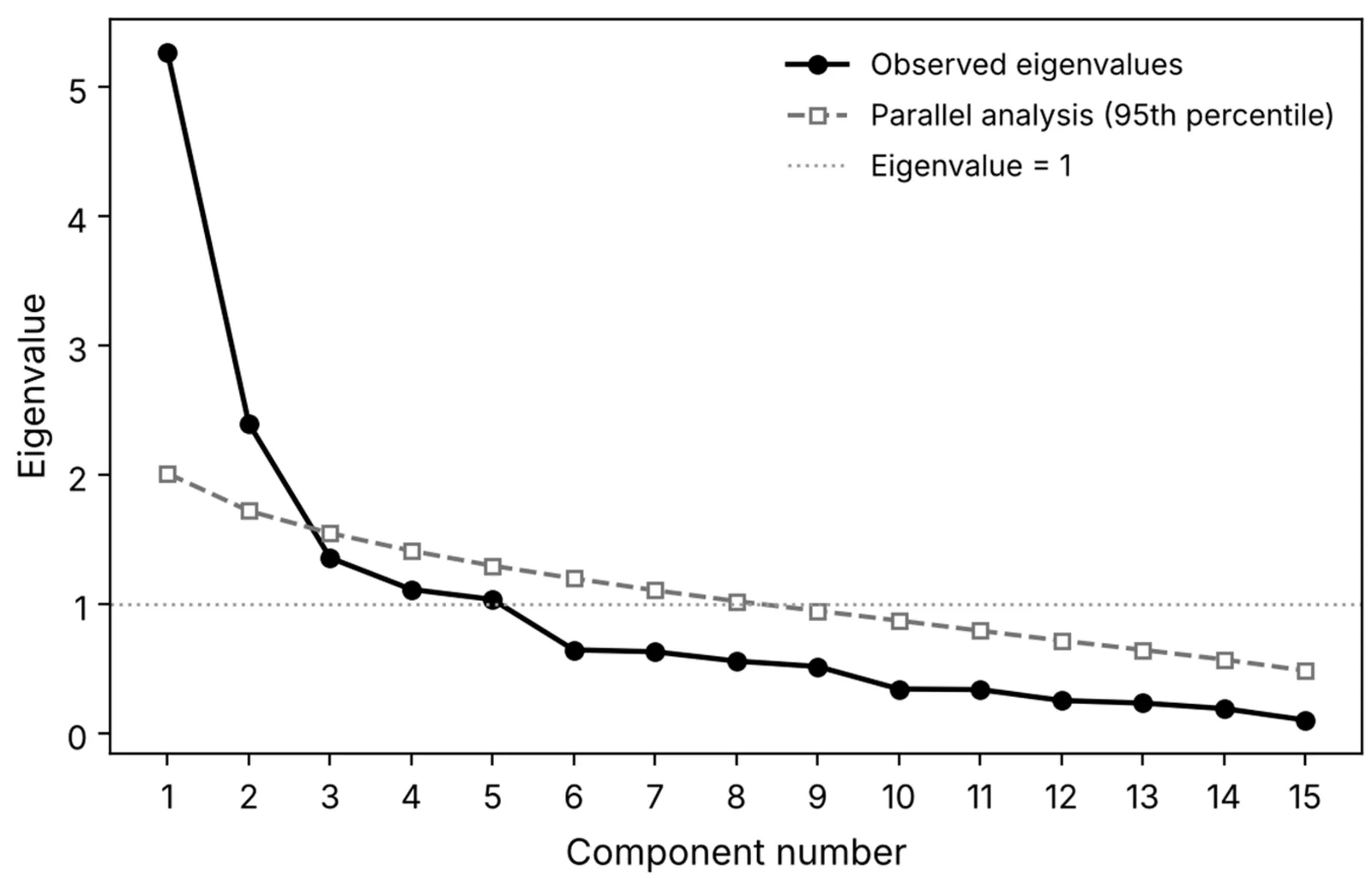
Scree plot for the 15-item g-SDQ principal component analysis, with the parallel-analysis criterion (95th percentile of eigenvalues from 2000 permuted datasets).

Sampling adequacy was acceptable (KMO = 0.73; item-level values 0.61–0.84), and Bartlett’s test of sphericity was significant (χ^2^(105) = 651.6, *p* < 0.001). Although five components had eigenvalues > 1.0 (5.27, 2.40, 1.36, 1.11, 1.04), parallel analysis indicated that only the first two exceeded the 95th percentile of the eigenvalues obtained from permuted data (2.01, 1.72, 1.55, 1.41, 1.30), and Velicer’s MAP test also reached its minimum at two components (Figure 2). A two-component solution was therefore retained on empirical grounds and subjected to Varimax rotation; together, the two components explained 51.1% of the total variance (Component 1: 27.6%; Component 2: 23.6%). The full rotated loading matrix and communalities (range 0.32–0.71) are presented in Table 4. Component 1 comprised SDQ1 (chewing difficulty, 0.837), SDQ10 (coughing with liquids, 0.817), SDQ2 (oral residue, 0.787), SDQ15 (respiratory infection, 0.739), SDQ7 (solid food sticking in the throat, 0.686), SDQ11 (coughing with solids, 0.572), and SDQ12 (voice change after swallowing, 0.564). Component 2 comprised SDQ3 (nasal regurgitation, 0.795), SDQ8 (difficulty with pureed food, 0.735), SDQ14 (breathing difficulty during meals, 0.698), SDQ13 (saliva aspiration, 0.645), SDQ4 (food dripping from the mouth, 0.641), and SDQ9 (sensation of a lump in the throat, 0.627), while SDQ5 (excess saliva/drooling; 0.448 vs. 0.376) and SDQ6 (repeated swallowing; 0.432 vs. 0.398) cross-loaded on both components. Thus, each component contained both oral- and pharyngeal-phase items, and the empirical structure did not reproduce the theoretical oral/pharyngeal division. Rather, Component 1 grouped the more frequently endorsed mealtime symptoms of bolus preparation and coughing (mean item endorsement 18.5%), whereas Component 2 grouped less frequently endorsed symptoms of bolus control, saliva management, and breathing (mean endorsement 9.1%); the components were therefore labelled “frequent mealtime symptoms” and “infrequent bolus-control and airway symptoms”, respectively. The sensitivity analysis with principal axis factoring and oblimin rotation reproduced the same item grouping (inter-factor correlation = 0.34). Cronbach’s α was 0.857 (95% CI 0.806–0.898) for Component 1 and 0.777 (0.699–0.841) for Component 2, and 0.674 (0.552–0.771) and 0.763 (0.681–0.831) for the theoretical oral (items 1–5) and pharyngeal (items 6–14; item 15 excluded due to its dichotomous scoring) subscales, respectively.

### 3.6. Concurrent and Construct Validity

Spearman correlations between the g-SDQ total score and the other clinical measures are shown in Table 5. Apart from phVF, cVF, and age, all comparators deviated significantly from normality (Shapiro–Wilk *p* < 0.05). Convergent validity was supported by strong positive correlations of the g-SDQ with both the DYMUS (ρ = 0.722, 95% CI 0.587–0.818) and the SMS (ρ = 0.722, 0.587–0.818; both FDR-adjusted *p* < 0.001). Construct validity was supported by a weak but significant positive correlation with the EDSS (ρ = 0.250, 0.039–0.439; FDR-adjusted *p* = 0.048), consistent with an association between neurological disability and self-reported swallowing difficulty. The g-SDQ scores did not correlate significantly with age (ρ = 0.094) or disease duration (ρ = 0.120).

For cognitive and language measures, the g-SDQ correlated negatively with phVF (ρ = −0.301, 95% CI −0.484 to −0.093; FDR-adjusted *p* = 0.014), indicating that greater self-reported dysphagia coincided with poorer phonemic fluency. Weak negative correlations with cVF (ρ = −0.185), the SDMT (ρ = −0.153), and the GVLT-II (ρ = −0.159) did not remain significant after FDR correction (adjusted *p* = 0.168–0.227), and no association was observed with the BVMT-R (ρ = 0.065). In the Pearson sensitivity analysis, the correlations with cVF, the SDMT, and the GVLT-II were nominally significant and survived FDR correction (Table 5); however, given the floor effect and right skew of the g-SDQ distribution, the Pearson coefficients are more strongly influenced by the small number of high-scoring participants and were not used for inference.

Regarding objective swallowing screening, pwMS with a positive 3oz-WST had significantly higher g-SDQ scores (median 11.0, IQR 8.75–12.25; mean 10.14 ± 4.88) than those with a negative 3oz-WST (median 0.5, IQR 0.5–2.5; mean 1.97 ± 2.64; Mann–Whitney U = 944.5, Z = 5.26, *p* < 0.001, r = 0.56).

## 4. Discussion

Dysphagia in MS meaningfully reduces patients’ quality of life and can lead to life-threatening complications, including aspiration pneumonia and even death [15]. Prompt screening is therefore essential to enable timely referral and treatment. The present study aimed to translate, culturally adapt, and psychometrically validate the g-SDQ in pwMS. To the best of our knowledge, this is the first study to validate the SDQ for Greek-speaking pwMS, addressing a clinically important gap in a country with a high and growing MS prevalence [23].

The g-SDQ demonstrated good internal consistency (α = 0.857; 95% CI 0.809–0.897), closely mirroring the reliability reported in the English and the Italian validation studies [20,21]. Item analysis supported the retention of all 15 items, since no removal produced a meaningful gain in α, thereby preserving the instrument’s full clinical coverage. However, the structural findings do not support separate oral and pharyngeal subscales. The theoretical oral subscale showed only questionable reliability (α = 0.674), and although parallel analysis supported two components, each contained items from both swallowing phases. Instead, the components appeared to separate frequently from infrequently endorsed symptoms, a pattern that may partly reflect differences in item endorsement rates in highly skewed ordinal data (so-called difficulty factors) rather than distinct clinical constructs. Although MS-related neurological impairment can affect the coordination of both oral and pharyngeal musculature [45], self-reported symptoms in our predominantly mildly disabled sample did not cluster by swallowing phase. Together with the dominant first eigenvalue (5.27, more than twice the second), these findings favour the use of the g-SDQ total score rather than phase-specific subscale scores; the dimensional structure should be re-examined in larger samples, ideally with confirmatory methods.

A key finding of this study is the tool’s promising discriminative validity, evidenced by an AUC of 0.912 (95% CI 0.791–0.990). Using the 3oz-WST as the clinical screening criterion, the optimal cut-off score of 6.5 provides a balanced trade-off between sensitivity and specificity. Bootstrap internal validation suggested only modest optimism in these estimates (optimism-corrected sensitivity 81.6% and specificity 93.2%); nevertheless, the optimal threshold varied across resamples (most often between 6.5 and 9.5), so the ≥6.5 cut-off should be regarded as provisional until externally validated. Notably, the previously proposed threshold of ≥11 [21] was highly specific (97.3%) but identified only half of the WST-positive participants in our cohort. This difference may reflect the use of a different reference standard (instrumental FEES-based assessment in the original Parkinson’s disease cohort [24] versus a bedside water-swallow screen in the present study), as well as differences in population and disease severity (a predominantly mildly disabled MS sample with a median EDSS of 3.0). For screening purposes, in which missed cases carry a greater clinical cost than false-positive referrals, a lower threshold appears preferable for Greek-speaking pwMS. Additionally, applying the stratification framework by Sparaco et al. [20] yielded consistent risk classifications; the vast majority of participants with a positive 3oz-WST were correctly classified as high-risk, whereas only one participant with the minimum SDQ score failed the 3oz-WST, suggesting that the SDQ total score distinguishes reasonably well between participants with and without screening indicators of dysphagia risk. However, given the small subset of WST-positive participants (*n* = 14), the resulting metrics carry inherent uncertainty (e.g., 95% CI for sensitivity 57.2–98.2%), underscoring the need for cautious clinical implementation and further validation in larger cohorts.

A substantial floor effect was also observed, with 52.3% of participants obtaining the minimum possible score, well above the 15% criterion [31]. This is not unexpected for a symptom-screening instrument administered to a predominantly mildly disabled outpatient sample, most of whom are asymptomatic, and it did not prevent the g-SDQ from separating WST-positive from WST-negative participants. Nevertheless, it implies a limited ability to differentiate among individuals with absent or very mild symptoms and may restrict sensitivity to change at the lower end of the scale, which should be considered if the instrument is used for follow-up assessments.

The weak positive correlation of the g-SDQ with the EDSS (ρ = 0.250) suggests that subjective dysphagia tends to be greater in pwMS with more advanced neurological disability, possibly reflecting progressive axonal loss within the brainstem and corticobulbar pathways [11], while the near-zero correlations with age and disease duration indicate that current neurological status may be more closely associated with subjective dysphagia symptoms than disease duration, aligning with the literature [46]. On the other hand, the strong correlations with DYMUS and SMS are clinically important; they indicate that the g-SDQ converges well with the two PROMs already validated for Greek pwMS and widely used in routine clinical care. Specifically, the internal consistency of the g-SDQ (α = 0.857) is closely comparable to that of the Greek DYMUS (α = 0.866), while adding symptom-frequency scoring, saliva- and pureed-food items, and recent respiratory events—content that the dichotomous solid/liquid format of DYMUS does not capture [20]. Similarly, it offers a clear complementary advantage over the SMS, which is a broader speech, voice, and swallowing instrument in which only a subset of items targets swallowing.

After correction for multiple comparisons, only phonemic VF remained significantly associated with the g-SDQ (*ρ* = −0.301), whereas the weak negative correlations with the SDMT, the GVLT-II, and cVF did not survive FDR correction, and no association was found with the BVMT-R. These findings should therefore be regarded as exploratory and hypothesis-generating and must be interpreted with caution. One possible interpretation is that the domains engaged by phonemic fluency (executive control, processing speed, and language) may align with the same distributed frontostriatal and white-matter networks implicated in the voluntary control of swallowing [47,48,49]. Cognitive dysfunction may also amplify perceived difficulty at meals through poorer self-monitoring, reduced dual-task capacity, and weaker adherence to compensatory strategies [50]. If confirmed in larger samples, these associations would support considering concurrent cognitive screening (e.g., BICAMS) in pwMS with a positive g-SDQ screen, and vice versa.

Nevertheless, several limitations should be acknowledged. First, the study is constrained by the lack of a pre-established sample size calculation, and the sample size restricts a factor analytical assessment, warranting validation in larger cohorts. Moreover, only 14 participants had a positive 3oz-WST; consequently, the sensitivity and PPV estimates are imprecise (e.g., 95% CI for sensitivity 57.2–98.2%), and the proposed cut-off requires external validation in independent samples. Another limitation is that the sample was unevenly distributed across MS phenotypes, with a large majority of RRMS (*n* = 61) and an under-representation of progressive forms (SPMS: *n* = 8 and PPMS: *n* = 0); phenotype data were also missing for a subset of participants due to inaccessible primary historical medical records at recruitment. Since dysphagia severity typically increases in progressive disease, future work should test the g-SDQ in larger and more phenotypically balanced samples. Additionally, although the 3oz-WST is a validated clinical screening test, it is limited in its ability to detect silent aspiration; the study did not include instrumental confirmation with FEES or VFSS, which remain the gold standard. Furthermore, the SDQ8 (difficulty with pureed food) and the SDQ14 (breathing difficulty during meals) items exhibited low endorsement rates in our sample (SDQ8 was endorsed by a single participant and had zero variance in approximately one-third of bootstrap resamples); consequently, the factor loadings associated with these items should be interpreted with caution; the same applies to the component structure as a whole. Finally, the cross-sectional design does not allow evaluation of the g-SDQ’s test–retest reliability or its responsiveness to change over time, both of which are important for longitudinal monitoring and treatment-response assessment.

## 5. Conclusions

In conclusion, this study provides a promising validation of the g-SDQ in pwMS. The instrument demonstrated good internal consistency and encouraging criterion-related and convergent validity relative to clinical screening parameters, with a provisional screening cut-off of ≥ 6.5 against the 3oz-WST. Its total score, rather than phase-specific subscale scores, is recommended for interpretation. Furthermore, it extends the clinical scope of existing tools such as the DYMUS by capturing symptom frequency and a broader range of dysphagic presentations. Consequently, the g-SDQ is recommended as a promising preliminary screening tool for identifying swallowing difficulties in MS patients, though further validation in larger prospective clinical cohorts is warranted before widespread implementation.

## Figures and Tables

**Table 1:** SDQ item-level frequencies: number and percentage of participants with scores ≥ 1, and breakdown by score value.

Item	N	%	Score 1	Score 2	Score 3
SDQ1	19	21.6%	10	8	1
SDQ2	16	18.2%	8	8	0
SDQ3	3	3.4%	3	0	0
SDQ4	7	8.0%	7	0	0
SDQ5	15	17.0%	13	2	0
SDQ6	15	17.0%	10	5	0
SDQ7	18	20.5%	9	8	1
SDQ8	1	1.1%	0	1	0
SDQ9	10	11.4%	8	2	0
SDQ10	23	26.1%	14	8	1
SDQ11	15	17.0%	11	4	0
SDQ12	12	13.6%	11	1	0
SDQ13	9	10.2%	8	1	0
SDQ14	4	4.5%	3	1	0
SDQ15	11	12.5%	11	—	—

Note. N and % refer to participants reporting a score ≥ 1 on the corresponding item. Score 1 = ‘rarely’ (≥once per month); Score 2 = ‘often’ (1–7 times per week); Score 3 = ‘very often’ (>7 times per week). Item SDQ15 uses a yes/no format; the “Score 1”column indicates affirmative responses (scored 2.5), while negative responses are scored 0.5. Dashes (—) denote response categories that do not apply to this item.

**Table 2:** SDQ: corrected item-total correlations and Cronbach’s α if item deleted.

Item	Corrected Item—Total Correlation	Cronbach’s α if Item Deleted
SDQ1	0.686	0.836
SDQ2	0.722	0.833
SDQ3	0.369	0.856
SDQ4	0.427	0.853
SDQ5	0.478	0.849
SDQ6	0.489	0.849
SDQ7	0.650	0.839
SDQ8	0.391	0.855
SDQ9	0.539	0.847
SDQ10	0.670	0.837
SDQ11	0.553	0.845
SDQ12	0.424	0.852
SDQ13	0.331	0.855
SDQ14	0.279	0.857
SDQ15	0.457	0.852

**Table 3:** Screening performance of g-SDQ cut-off scores against the 3oz-WST (*n* = 88; 14 WST-positive).

Index	≥6.5 (Youden-Optimal)	≥11 (Previously Proposed [21])
TP/FN/FP/TN	12/2/5/69	7/7/2/72
Sensitivity, % (95% CI)	85.7 (57.2–98.2)	50.0 (23.0–77.0)
Specificity, % (95% CI)	93.2 (84.9–97.8)	97.3 (90.6–99.7)
PPV, % (95% CI)	70.6 (44.0–89.7)	77.8 (40.0–97.2)
NPV, % (95% CI)	97.2 (90.2–99.7)	91.1 (82.6–96.4)
LR+ (95% CI)	12.69 (5.30–30.37)	18.50 (4.28–79.98)
LR− (95% CI)	0.15 (0.04–0.55)	0.51 (0.30–0.87)
Youden’s J	0.790	0.473

Note. TP = true positives; FN = false negatives; FP = false positives; TN = true negatives; PPV = positive predictive value; NPV = negative predictive value; LR = likelihood ratio. The 95% CIs for proportions are exact (Clopper–Pearson); CIs for likelihood ratios were computed on the log scale. Because all total scores end in 0.5, the ≥11 threshold corresponds to ≥11.5. Predictive values depend on the proportion of WST-positive participants in this sample (15.9%). Bootstrap optimism-corrected sensitivity and specificity for the ≥6.5 cut-off were 81.6% and 93.2%, respectively.

**Table 4:** Varimax-rotated loadings of the two-component principal component solution and communalities (h^2^) for the 15 g-SDQ items.

Item	Content	Endorsed, %	Component 1	Component 2	h^2^
SDQ1	Chewing difficulty	21.6	**0.837**	0.084	0.708
SDQ2	Food residue in the mouth	18.2	**0.787**	0.251	0.683
SDQ3	Food/liquid coming out of the nose	3.4	0.030	**0.795**	0.633
SDQ4	Food dripping from the mouth	8.0	0.211	**0.641**	0.456
SDQ5	Excess saliva/drooling	17.0	0.376	**0.448**	0.342
SDQ6	Repeated swallowing of chewed food	17.0	0.398	**0.432**	0.346
SDQ7	Solid food sticking in the throat	20.5	**0.686**	0.277	0.547
SDQ8	Difficulty swallowing pureed food	1.1	0.100	**0.735**	0.551
SDQ9	Sensation of a lump in the throat	11.4	0.350	**0.627**	0.516
SDQ10	Coughing when swallowing liquids	26.1	**0.817**	0.076	0.673
SDQ11	Coughing when swallowing solids	17.0	**0.572**	0.272	0.401
SDQ12	Voice change after eating or drinking	13.6	**0.564**	0.049	0.320
SDQ13	Coughing/breathing difficulty due to saliva	10.2	0.078	**0.645**	0.422
SDQ14	Breathing difficulty during meals	4.5	−0.008	**0.698**	0.487
SDQ15	Respiratory infection in the past year	12.5	**0.739**	−0.187	0.581
	Sum of squared loadings		4.13	3.53	
	% of variance		27.6	23.6	51.1

Note. Loadings ≥ 0.40 are shown in bold. Signs were reflected so that positive loadings indicate greater symptom burden. Component 1 = frequent mealtime symptoms; Component 2 = infrequent bolus-control and airway symptoms. Endorsed = percentage of participants scoring ≥1 on the item (Table 1). KMO = 0.73; Bartlett’s χ^2^(105) = 651.6, *p* < 0.001.

**Table 5:** Spearman (primary analysis) and Pearson (sensitivity analysis) correlations between the g-SDQ total score and clinical measures, with Benjamini–Hochberg FDR-adjusted *p*-values. Correlations significant after FDR correction are shown in bold.

Variable	*n*	Spearman ρ (95% CI)	*p*	*p* (FDR)	Pearson r	*p* (FDR)
**DYMUS**	**88**	**0.722 (0.587, 0.818)**	**<0.001**	**<0.001**	0.824	<0.001
**SMS**	**88**	**0.722 (0.587, 0.818)**	**<0.001**	**<0.001**	0.764	<0.001
**EDSS**	**88**	**0.250 (0.039, 0.439)**	**0.019**	**0.048**	0.458	<0.001
**phVF**	**88**	**−0.301 (−0.484, −0.093)**	**0.004**	**0.014**	−0.334	0.004
cVF	88	−0.185 (−0.382, 0.027)	0.084	0.168	−0.274	0.018
SDMT	86	−0.153 (−0.355, 0.062)	0.159	0.227	−0.249	0.030
GVLT-II	88	−0.159 (−0.357, 0.054)	0.140	0.227	−0.271	0.018
BVMT-R	77	0.065 (−0.161, 0.285)	0.573	0.573	0.002	0.988
Age	88	0.094 (−0.119, 0.298)	0.385	0.428	0.109	0.378
Disease duration	77	0.120 (−0.108, 0.335)	0.300	0.375	0.110	0.378

Note. ρ = Spearman’s rank correlation coefficient; CI = confidence interval (Fisher z-transformation with the Bonett–Wright standard error); FDR = Benjamini–Hochberg false discovery rate adjustment across the ten correlations; DYMUS = Dysphagia in Multiple Sclerosis questionnaire; EDSS = Expanded Disability Status Scale; SMS = Speech Pathology-Specific Questionnaire for Persons with Multiple Sclerosis; phVF = phonemic Verbal Fluency; cVF = categorical Verbal Fluency; SDMT = Symbol Digit Modalities Test; GVLT-II = Greek Verbal Learning Test–II; BVMT-R = Brief Visuospatial Memory Test–Revised.

## Data Availability

The data underlying this article will be shared on reasonable request to the corresponding author.

## Abbreviations

The following abbreviations are used in this manuscript:

MS	Multiple sclerosis
RRMS	Relapsing-remitting multiple sclerosis
SPMS	Secondary progressive multiple sclerosis
PPMS	Primary Progressive multiple sclerosis
pwMS	Persons with multiple sclerosis
FEES	Fibreoptic endoscopic evaluation of swallowing
VFSS	Videofluoroscopic swallowing study
PROM	Patient-reported outcome measure
DYMUS	Dysphagia in multiple sclerosis
SDQ	Swallowing disturbance questionnaire
SMS	Speech Pathology-Specific Questionnaire for Persons with Multiple Sclerosis
3-oz WST	3-ounce Water Swallow Test
BICAMS	Brief International Cognitive Assessment for Multiple Sclerosis
SDMT	Symbol digit modalities test
GVLT	Greek Verbal Learning Test
BVMT-R	Brief visuospatial memory test-revised
VF	Verbal fluency
phVF	Phonemic verbal fluency
cVF	Categorical verbal fluency
EDSS	Expanded Disability Status Scale
SD	Standard deviation
AUC	Area under the curve
CI	Confidence interval
FDR	False discovery rate
KMO	Kaiser–Meyer–Olkin measure of sampling adequacy
LR	Likelihood ratio
MAP	Minimum average partial test
NPV	Negative predictive value
PCA	Principal component analysis
PPV	Positive predictive value
ROC	Receiver operating characteristic
